# Endometriosis – novel approaches and controversies debated

**DOI:** 10.1530/RAF-21-0097

**Published:** 2021-11-05

**Authors:** W G Foster, M Leonardi

**Affiliations:** 1 Department of Obstetrics & Gynecology, McMaster University, Hamilton, Ontario, Canada

Defined by the extrauterine growth of estrogen-dependent endometrial-like epithelial and stromal cells, endometriosis is a common gynecological and systemic inflammatory disease affecting approximately 179 million people assigned female at birth (predominately cisgender women) worldwide. Although most frequently detected in the pelvic cavity, endometriotic lesions can be found throughout the body. Three main phenotypes include endometriomas, superficial, and deep endometriosis. Lesion appearance is variable and dependent on the tissue on which it grows. Hallmark features of endometriosis include pelvic pain and infertility; however, some people with endometriosis remain asymptomatic. Endometriosis is a disease whose impact on the health care system exceeds that of caring for women with Crohn’s disease, asthma, migraines, and rheumatoid arthritis (Simoens* et al.* 2007, 2011, 2012, Klein* et al.* 2014). Although a relatively common disease with a high economic burden, endometriosis remains underfunded and under-researched (As-Sanie* et al.* 2019). While important advances have been made over the years in defining the pathophysiology of endometriosis, the cause of endometriosis remains ill-defined, diagnosis continues to present challenges and therapeutic options are suboptimal. Patients frequently report dissatisfaction with current therapeutic options prompting the search for alternative treatments including non-hormonal alternatives. In a special series that will be running in *Reproduction and Fertility* over the coming months, international experts have been recruited to provide insights and perspectives into the latest advances in endometriosis research and treatment. We strive to succeed with this special series in summarizing the current state of ‘leading edge’ research and opinion in endometriosis. Though not exhaustive, the topics and authors capture this moment in time in endometriosis research.

Although widely recognized to be an estrogen-dependent disease, numerous physiological pathways are known to be dysregulated in people with endometriosis including cell adherence, attachment, proliferation, apoptosis, angiogenesis, and tissue remodeling enzyme expression (Hey-Cunningham* et al.* 2013). That endometriosis may have a heritable component is not a new concept (Saha* et al.* 2015); however, specific gene mutations and gene regulation continue to be explored. Indeed, the mechanisms regulating different pathways dysregulated in endometriotic tissues are beginning to be teased apart with increasing attention focused on mechanisms regulating gene expression including chromatin architecture, long ncRNA, micro-RNA, and piwi-RNA. The role of gonadal steroids in modulating the expression of epigenetic regulators of gene expression in endometriosis is poorly understood. Early in this special series, the relationship between gonadal steroids and genomic regulation is reviewed by Dr Philippa Saunders. Given the prominent role of estrogen and the use of androgens as a therapeutic option in endometriosis suggests that hormone replacement therapy is a potential modifying factor in the transgender population that is beginning to receive attention. The prevalence of endometriosis and its implication in transgender men is summarized by Dr Cecile Ferrando in her review of this underserved population.

The reproductive and gastrointestinal tract microbiome has been described by several investigators and dysbiosis has been linked with endometriosis (Franasiak* et al.* 2016, Laschke & Menger 2016, Hernandes* et al.* 2020, Leonardi* et al.* 2020*a*). In addition, the role of the microbiome in disease, cancer, and modulating behavior has received increasing attention. Dr Mauricio Abrão reviews recent advances in the microbiome and its potential role in the pathogenesis of endometriosis. Dysregulation of the immune function and inflammation are well known in patients with endometriosis. Moreover, chronic inflammatory conditions are linked with increased risk of cardiovascular disease and stroke (Appelman* et al.* 2015) prompting interest in health sequelae arising from endometriosis (Mu* et al.* 2016). Elucidating the long-term health consequences of endometriosis will be discussed by Dr Stacey Missmer in her review.

Arriving at a diagnosis of endometriosis continues to challenge both patients and health care providers. Challenges in arriving at a diagnosis of endometriosis include early age at onset of symptoms, normalization of pain, and symptom suppression through intermittent use of oral contraceptive pills (Ballard* et al.* 2006, Nnoaham* et al.* 2011). Though actively being challenged, the gold standard for diagnosis remains laparoscopic visualization and histological confirmation of endometriotic implants, with diagnostic delays of 5.3–12 years from the onset of the first symptom to surgical diagnosis (Simoens* et al.* 2007, 2012, Singh* et al.* 2020). Although risks to patients from laparoscopy are rare, they are significant if they occur (Slack* et al.* 2007) and patients with endometriosis can expect to undergo multiple diagnostic and operative laparoscopies over the course of their disease (Jarrell 2010, Agarwal* et al.* 2021). Diagnostic delay, cost, surgical risk, and poor correlation between symptoms and extent of disease are the basis for arguments to shift away from a surgical diagnosis (Taylor* et al.* 2018, Agarwal* et al.* 2019). Ideally, a diagnosis can be achieved in an accurate and reliable manner, with non-invasive imaging providing the most optimistic method to visualize disease directly. Recent advances in ultrasound and MRI techniques have brought the diagnosis of endometriomas and deep endometriosis into the realm of possibility. In this special series, Dr Stephano Guerriero will be providing an overview of recent advances and emerging techniques. However, diagnosis remains elusive to many currently and imaging may not be a panacea. Thus, there is an urgent unmet need to identify novel clinical markers of endometriosis (Nisenblat* et al.* 2016, Rogers* et al.* 2017, Agarwal* et al.* 2019).

Those with endometriosis report dissatisfaction with their care, and treatment options remain suboptimal. Historically, endometriosis has been treated in acute care or surgical model; however, persistent pelvic pain and recurrence of disease and/or pain following surgical removal of lesions brings attention to the need to readdress the current approach to care. Specifically, Dr Sanjay Agarwal introduces the concept of a chronic care model for the management of people with endometriosis. Alongside the changing model of care, there is an obvious need for novel medical treatments, especially in the non-hormonal category, which is being reviewed by Dr Hiroshi Kobayashi in this issue. Alternatives to current medical and surgical treatment options are also receiving attention (Leonardi* et al.* 2020*b*). Environmental factors and diet are well-established modifiers of health and disease; however, the role of diet as a therapeutic option in endometriosis is emerging as a potential option worthy of consideration. In this series, Dr Annemiek Nap will be discussing the role of diet in endometriosis as a novel approach to managing endometriosis symptoms. Environmental conditions and diet also differ across racial groups. The impact of race on disease extends beyond diet; understanding the prevalence of endometriosis among racial groups and diagnostic and treatment considerations is an issue explored by Dr Olga Bougie.

Finally, we will be concluding this special series with a debate presenting opposing viewpoints on the future of diagnostic laparoscopy as a *diagnostic test*. Arguments in favor of diagnostic laparoscopy will be presented by Dr George Condous and the contrary arguments presented by Dr Kelly Wright. The role of diagnostic laparoscopy as a stand-alone diagnostic test for endometriosis has become controversial in recent years, often being used at the same time as operative laparoscopy for the surgical treatment of endometriosis. While many advocate for a reduction in the number of laparoscopies in favor of a clinical diagnosis and presumptive management of disease, others maintain that laparoscopy is a valuable tool that should remain prominent in the diagnosis of people with endometriosis.

## Declaration of interest

The authors declare that there is no conflict of interest that could be perceived as prejudicing the impartiality of this commentary.

## Funding

This work did not receive any specific grant from any funding agency in the public, commercial or not-for-profit sector.

## Author contribution statement

Both authors jointly conceived of the concepts for this manuscript, contributed equally to the written document, and jointly approved the final manuscript for submission.
